# Glutathione S-transferases polymorphisms confer susceptibility to senile cortical cataract in the Han Chinese population

**Published:** 2012-05-11

**Authors:** Zhengxuan Jiang, Kun Liang, Qing Zhang, Liming Tao

**Affiliations:** Department of Ophthalmology, the Second Affiliated Hospital of Anhui Medical University, Hefei, P.R. China

## Abstract

**Purpose:**

The aim of this study was to examine whether glutathione S-transferase (*GST*) polymorphisms were associated with a susceptibility to age-related cortical cataract (cortical ARC) in the Han Chinese population.

**Methods:**

Glutathione S-transferase M1 (*GSTM1*) and glutathione S-transferase T1 (*GSTT1*) gene polymorphisms were genotyped in 422 Han Chinese patients with age-related cortical cataract, and in 312 age, sex, and ethnically matched healthy controls, using a multiplex polymerase chain reaction.

**Results:**

The results showed that the *GSTM1* positive genotype had an increased risk of developing cortical ARC (p=0.0002, odds ratio [OR] 1.74, 95% CI 1.30 to 2.34). There was a statistically significant association between the *GSTM1* positive genotype and the risk of cataract development in both female and male groups (p=0.026, OR 1.58, 95% CI 1.05 to 2.36; p=0. 002, OR 1.97, 95% CI 1.27 to 3.04, respectively). A combination of *GSTM1* positive and *GSTT1* null genotypes was associated with the risk of developing age-related cortical cataract (p=0.002, OR 2.19, 95% CI 1.33 to 3.60). The results revealed that the *GSTM1* positive genotype was significantly higher in the smoker patients group as compared to the non-smoker patients group (p=0. 016, OR 1.62, 95% CI 1.09 to 2.39). Logistic regression analysis revealed that smoking may be a risk factor for the development of ARC (r=0.120, p=0.013).

**Conclusions:**

Our study suggests that the *GSTM1* positive genotype and a combination of *GSTM1* positive and *GSTT1* null genotypes may be associated with a susceptibility to age-related cortical cataract in the Han Chinese population. The current study indicates that smoking may be an important factor in the development of cortical ARC.

## Introduction

Cataract is one of the leading causes of visual impairment and blindness in the world today [1]. It is estimated that cataract affects approximately 37 million people and accounts for 48% of blindness worldwide [2]. Age-related cataract (ARC) accounts for about 80% of all cataracts [1]. ARC is classified as nuclear, cortical, or posterior sub-capsular cataract, according to the location of the opacity within the lens [3]. Although the pathogenesis of ARC is not fully understood, many studies suggest that genetic factors and oxidative stress are two major factors in the development of age-related cataract [4]. Family-based linkage studies reported multiple ARC loci at 1q31, 2p24, 2q11, 4q28, 15q13, and 6p12-q12 [5]. Moreover, it is known that among the four types of ARC, cortical cataract is highly heritable [6,7]. Previous studies have identified several genes to be associated with ARC, such as heat shock protein (*HSF4*), gap junction protein alpha 8 (*GJA8*), eph-receptor tyrosinekinase-type A2 (*EPHA2*), and glutathione S-transferase (*GST*). However, these genes account only partially for the genetic predisposition to ARC. Studies need to be performed to search for other genes, related to oxidative stress, which may be responsible for the development of ARC.

Glutathione S-transferases (*GSTs*) are a group of dimeric detoxification enzymes that play an important role in detoxifying exogenous and endogenous toxic compounds under conditions of oxidative stress [8-11]. The GST isoenzymes exist in human tissue, and human lens tissue has been reported to express class mu, theta, and pi [12]. Glutathione S-transferase M1 (*GSTM1*) and glutathione S-transferase T1 (*GSTT1*) have been found to have functional polymorphisms and have been studied extensively in relation to disease [11,13-15]. As the genetic factor is a major cause of cortical ARC, cortical ARC patients were selected as subjects for this study. The study was designed to examine whether there is an association of *GST* polymorphisms with susceptibility to cortical ARC in the Han Chinese population.

## Methods

### Patients and healthy controls

A total of 422 age-related cortical cataract patients and 312 age, sex, and ethnically matched healthy controls were recruited from The Second Affiliated Hospital of Anhui Medical University (Anhui, P.R. China). All the patients and healthy controls underwent a full ophthalmic examination, including visual acuity, slit lamp examination of the anterior chamber, gonioscopy, central corneal thickness, measurement of intraocular pressure, fundus examination with special attention paid to optic disc parameters, and visual field. The degree of cataract in all patient eyes was either CII or CIII, according to the Lens Opacities Classification System, version II (LOCS II) [16]. Subjects were selected who were free of other ocular disease or systemic diseases. Information on hypertension, diabetes mellitus, prolonged corticosteroid administration, and other known causes of cataract was excluded from this study. All subjects were interviewed to get data on their smoking habits. The study was approved by the local institutional ethics committee of The Second Affiliated Hospital of Anhui Medical University. All procedures followed the tenets of the Declaration of Helsinki. Written, informed consent was obtained from all the subjects. After obtaining written, informed consent, we took 5 ml of peripheral blood from each participant. Blood samples were collected in EDTA tubes and kept at −70 °C until use. Genomic DNA was extracted from peripheral blood by the QIAamp DNA Blood Mini Kit (Qiagen, Hilden, Germany).

### Genotyping

The *GSTM1* and *GSTT1* polymorphisms were genotyped using the multiplex polymerase chain reaction (PCR). The PCR primers used in present study were as described [17]. Primers for *GSTM1* were 5′-GAA CTC CCT GAA AAG CTA AAG C-3′ and 5′-GTT GGG CTC AAA TAT ACG GTG G-3′. Primers for *GSTT1* were 5′-TTC CTT ACT GGT CCT CAC ATC TC-3′ and 5′-TCA CCG GAT CAT GGC CAG CA-3′. To avoid false- negative results, the β-globin gene was used as an internal control. Primers for β-globin were 5′-CAA CTT CAT CCA CGT TCA CC-3′ and 5′-GAA GAG CCA AGG ACA GGT AC-3′. Each PCR reaction was performed in a 10-μl reaction mixture containing 5 μl Premix Taq (Ex Taq Version; TaKaRa Biotechnology Co. Ltd., Dalian, China), 15 pmol primers, and 0.1 μg of genomic DNA for ampliﬁcation of the DNA. The conditions of PCR were as follows: Amplification was performed by initial denaturation at 94 °C for 5 min, followed by 33 cycles at 94 °C for 40 s, at 64 °C for 40 s, and at 72 °C for 40 s, with a final extension at 72 °C for 5 min.

Digestion products were visualized on a 2.0% agarose gel, and were stained with GoldView (SBS Genetech, Beijing, China). The product lengths for *GSTM1*, *GSTT1*, and β-globin were 219 bp, 459 bp, and 268 bp, respectively. In the presence of the β-globin band condition, an absence of PCR product for *GSTM1* or *GSTT1* was described as *GSTM1* null or *GSTT1* null genotype.

### Statistical analysis

Student’s *t*-test was used to compare the ages of the patient and control groups. The *GSTM1* and *GSTT1* genotypes were compared between patients and controls by the χ^2^ test, using SPSS (version 10.0; SPSS Inc., Chicago, IL). Logistic regression analysis was used to estimate the actual risk conferred by the genetic variants (version 10.0; SPSS Inc.). The p-values were corrected (Pc) with the Bonferroni correction by multiple comparison with the number of analyses performed. Statistical significance was set at a p value <0.05.

## Results

Four hundred and twenty-two patients with age-related cortical cataracts and three hundred and twelve healthy controls were recruited for the present study. The average age of the cortical ARC patients was 59.61±9.52 years. The average age of the healthy controls was 57.34±10.27 years. There was no significant difference between patients and controls as regards age.

Distribution of the *GSTM1* and *GSTT1* genotypes in patients and healthy controls are shown in Table 1. The results show that the frequency of the *GSTM1* positive genotype is significantly higher in the patients relative to the healthy controls. The frequency of the *GSTT1* null/positive genotype was not significantly different between the patients and the healthy controls. We also investigated the association between a combination of *GST* genotypes and cortical ARC. The results are shown in Table 2. It was found that the risk of cortical ARC was significantly increased in patients with a combination of *GSTM1* positive and *GSTT1* null genotypes.

**Table 1:** Frequencies of genotypes of *GST* polymorphisms in ARC patients and controls.

**Genotype**	**Sex**	**Patients with ARC**	**Controls**	**P_C_**	**OR (95% CI)**
***GSTM1***	**Both sex**				
Positive		246 (58.3%)	139 (44.6%)	0.0002	1.74 (1.30–2.34)
Null		176 (41.7%)	173 (55.4%)		
***GSTT1***					
Positive		201 (47.6%)	174 (55.8%)	0.186	1.22 (0.91–1.63)
Null		221 (52.4%)	138 (44.2%)		
***GSTM1***	**Male**				
Positive		122 (62.8%)	68 (46.3%)	0.002	1.97 (1.27–3.04)
Null		72 (37.2%)	79 (53.7%)		
***GSTT1***					
Positive		95 (48.9%)	83 (56.4%)	0.170	0.74 (0.48–1.14)
Null		99 (51.1%)	64 (43.6%)		
***GSTM1***	**Female**				
Positive		124 (54.4%)	71 (43.1%)	0.026	1.58 (1.05–2.36)
Null		104 (45.6%)	94 (56.9%)		
***GSTT1***					
Positive		106 (46.5%)	91 (55.1%)	0.090	0.71 (0.47–1.06)
Null		122 (53.5%)	74 (44.9%)		

GST, glutathione S-transferase; OR, odds ratio; Pc, Bonferroni corrected p value.

**Table 2:** Association between cortical cataract and combination of *GSTM1* and *GSTT1* genotypes.

***GSTM1***	***GSTT1***	**Patients with ARC (%)**	**Controls (%)**	**P_C_**	**OR (95% CI)**
Positive	Positive	109 (25.8%)	81 (26.0%)	0.008	1.76 (1.15–2.67)
Positive	Null	137 (32.5%)	58 (18.6%)		
Null	Positive	92 (21.8%)	93 (29.8%)		
Null	Null	84 (19.9%)	70 (22.4%)		

GST, glutathione S-transferase; OR, odds ratio; Pc, Bonferroni corrected p value.

We questioned whether the sex of the subjects can affect the *GST* genotype, so tested the association between *GST* genotype and cataract risk, stratified by sex. There was a statistically significant association between the *GSTM1* positive genotype and ARC in both female and male groups. The result revealed that in this study gender was not a risk factor for the development of ARC.

To search for risk factors in the development of ARC, we divided the patients’ samples into two groups according to smoking status. The results showed that the *GSTM1* positive genotype was significantly higher in the smoker patients’ group. The results were shown in the Table 3. Age and smoking status had reported to involve in the pathogenesis of ARC, Logistic regression analysis was used to estimate the actual risk conferred by the genetic variants. The result revealed that smoking may be a risk factor for the development of ARC (r=0.120, p=0.013). Table 4 shows these results.

**Table 3:** Association between smoking status and *GST* polymorphisms in the cortical cataract group.

**Genotype**	**Smoker group (%)**	**Nonsmoker group (%)**	**P_C_**	**OR (95% CI)**
***GSTM1***				
Positive	139	107	0.016	1.62 (1.09–2.39)
Null	78	97		
***GSTT1***				
Positive	102	99	0.791	0.95 (0.65–1.39)
Null	115	106		

GST, glutathione S-transferase; OR, odds ratio; Pc, Bonferroni corrected p value.

**Table 4:** Logistic regression analysis between age, smoking status and *GST* polymorphisms in the cortical cataract group.

**Factor**	**Wald**	**r**	**p-value**	**OR (95% CI)**
***GSTM1***				
Smoking	6.09	0.120	0.013	1.63 (1.10–2.41)
Age	0.00	0.001	0.983	1.00 (0.97–1.04)
***GSTT1***				
Smoking	1.58	0.043	0.787	0.96 (0.66–1.40)
Age	0.02	0.003	0.947	1.04 (0.93–1.12)

OR, odds ratio.

## Discussion

Age-related cataract is one of the most common causes of useful vision loss in the world [18]. Although the etiology and pathogenesis of ARC are still unclear, several hypotheses have been proposed, including an oxidative stress lesion and a genetic factor. Among the assumptions, the oxidative stress lesion mechanism has been considered the major cause of senile cataract. Thus, the lens must have an efficient detoxification system to protect it against the toxic effects of oxidative damage [8,10,19]. Recent biochemical studies have suggested that GSTs are important enzymes in defense against oxidative stress. However, they also play roles in reactions that result in toxic products which may cause structural alterations to the proteins [20]. As genetic polymorphisms may alter the function of these enzymes, many studies have attempted to show an association between *GST* polymorphic variants and disease susceptibility. The *GSTM1* and *GSTT1* polymorphisms were reported to be associated with many diseases, including glaucoma [21,22], acute leukemia [23], senile macular degeneration [24], and cataract, in the different populations tested [25]. These results stimulated us to test whether the polymorphisms of *GSTM1* and *GSTT1* could contribute to the development of cortical ARC in the Han Chinese population.

The present study suggests that the *GSTM1* positive genotype is associated with cortical ARC and may be a genetic risk factor for the development of this disease. These results agree with previous findings reporting on senile cortical cataract in the Estonian and Turkish populations [20,26]. However, the *GSTM1* null genotype has been reported to be associated with an increased risk of cataract in Asian populations [15,25]. The result showed that the *GSTT1* null genotype was not associated with cortical ARC. Similarly, no association was found between the *GSTT1* null genotype and cataract in Iranian [27], Turkish and Estonian populations [26,28]. In contrast, the *GSTT1* null genotype was reported to be associated with an increased risk for senile cataract in Asian populations, and with primary open-angle glaucoma (POAG) in a Turkish population [11,15]. The association between the genotype profile and cortical ARC was also tested. The results showed that a combination of *GSTM1* positive and *GSTT1* null genotypes was significantly associated with cortical ARC development. Similarly, a combination of GSTM1 positive and GSTT1 null genotypes was associated with the incidence of POAG in a Turkish population [11]. Many factors might explain the difference in results: First, the differences in the number of subjects in genetics studies may result in different outcomes [15]. Second, the patients in the other study included all types of senile cataracts, while our study involved only cortical cataracts. Furthermore, differences in the ethnic, genetic and environmental background of the subjects may have influenced the results. Subjects in our study all came from the Anhui province, which is in the central part of China, while the subjects of the other study were recruited from Beijing city, which is in the northern part of China [25].

There are several reasons to account for the association between the *GSTM1* positive genotype and cortical ARC. On the one hand, GST enzymes are very useful for detoxification and defense against oxidative stress, but they are also involved in reactions that result in toxic products. The toxic products may lead to structural damage of the proteins in the lens that then cause lens opacification [20,26]. On the other hand, the GST activity is significantly decreased in cataractous lens compared with that in normal lens, the *GSTM1* positive genotype may have lost its ability to detoxify the harmful substances under oxidative stress.

There are many clinical and life-style factors may influence the results of association study. In current study, the patients were divided into two groups according to smoking status. The *GSTM1* positive genotype was significantly higher in the smoker patients group. It may be assumed that smoking leads to an increase of toxic compounds in the lens tissue. The increased toxicity would then lead to damage of the lens protein [19,26]. As age and smoking may both be factors in the pathogenesis of ARC, logistic regression analysis was used to estimate the actual risk conferred by the genetic variants. The results suggest that smoking may be a risk factor in the development of ARC and may provide very useful information for preventing the ARC. Although some studies reported that the effect of gender differences on the association between *GST* polymorphisms and senile cataract, this study did not find any association between them. It was consistent with the result of Meta-analysis between *GST* polymorphisms and senile cortical cataract [15].

As with other candidate gene studies, there are several limitations inherent to our study. First, the subjects were recruited from a Han Chinese population only. The results need to be confirmed in other ethnic populations. Second, extensive research is needed to clarify how the *GST* genotypes influence susceptibility to cataract. Third, many recent publications have analyzed the single copy deletions in *GSTM1* and *GSTT1*, but this study has only tested the complete deletions in them, and not the single copy deletions. In addition, the patients in this study were only cortical cataract cases, other types of ARC should be tested in subsequent studies.

In summary, our study showed that the *GSTM1* positive genotype and a combination of *GSTM1* positive and *GSTT1* null genotypes were associated with susceptibility to cortical ARC. The predisposing effect of the *GSTM1* positive genotype to cortical ARC was stronger in the smoker patients group, and smoking may therefore be a risk factor for this disease. This study may give some information for diagnosis and preventing the ARC.
